# How does the double-track human resource management model contribute to job burnout and mental health among Chinese government departments? A Chinese police study

**DOI:** 10.3389/fpubh.2024.1423103

**Published:** 2024-09-05

**Authors:** Zhengyan Liang, Min Yao, Hao Li, Jiayu Chen, Mei Yang, Tian Tang, Hong Ye, Yuqing Zeng, Minqiang Zhang

**Affiliations:** ^1^Key Laboratory of Brain, Cognition and Education Sciences, Ministry of Education; Institute for Brain Research and Rehabilitation, and Guangdong Key Laboratory of Mental Health and Cognitive Science, South China Normal University, Guangzhou, China; ^2^School of Psychology, South China Normal University, Guangzhou, China

**Keywords:** double-track human resource management, job burnout, auxiliary police, job performance, mental health

## Abstract

**Objectives:**

This study aims to investigate the contribution of the double-track human resource management model to the job performance and mental health of frontline police within China’s public security organs.

**Methodology:**

An individual-centered approach, latent profile analysis (LPA), was utilized in this study, which used cluster sampling to survey all police of all 118 frontline police stations in an economically underdeveloped area of China and 839 personnel were selected for the analysis. This method allowed for a detailed examination of the contribution of the double-track system to job performance and mental health.

**Findings and conclusion:**

The study identified three subtypes of job burnout among Chinese police: low job burnout, medium job burnout, and emotional exhaustion type. The double-track human resource management model in China’s public security organs has contributed to significant disparities between civilian and auxiliary police, such as more severe job burnout among civilian police, lower job performance, and mental health among auxiliary police.

**Implications:**

To mitigate the potential risks associated with the double-track human resource management model, adjustments are necessary for both the management system and the treatment distribution system, which would also help address the disparities and improve the overall wellbeing and performance of all police officers.

## 1 Introduction

The double-track human resource management model represents a distinctive human resource management model within China’s governmental departments. This model encompasses two employment arrangements within one institution: the state permanent employment, which receives salaries and benefits funded by the government, and contract employment directly managed by the institution itself, primarily aimed at addressing human resource shortages. A significant disparity exists between these two arrangements related to salary, benefits, career advancement opportunities, and democratic rights within the institution. Notably, the police force stands as the most emblematic occupation under this management model.

As economic and societal development progresses, the widening gap between rich and poor has engendered various social challenges, including heightened security concerns, increased crime rates, and emergent group incidents (1). Concurrently, severe shortages in police personnel have emerged. To address these issues, police departments of China have openly recruited contract workers, called “auxiliary police,” to actively assist the civilian police in law enforcement, order maintenance, and service activities. In many frontline police departments, the number of auxiliary police exceeds that of the civilian police. Compared to civilian police, the auxiliary police are engaged in relatively hard work on the front line and receive lower salaries and welfare. Additionally, they encounter limited opportunities for career development, leading to severe job burnout and mental health (2, 3). In this context, this study aimed to explore how the double-track human resource management model contributes to job burnout and mental health among Chinese police.

## 2 Literature review

### 2.1 Job burnout and the relationship among its dimensions

Job burnout refers to the extreme psychological strain experienced by workers (e.g., police officers and nurses) in the human service professions (4). The definition proposed by Maslach and Jackson (5) is widely accepted, characterizing burnout as a three-dimensional syndrome affecting individuals primarily engaged in providing care or services to others. Burnout is expressed by emotional exhaustion (feeling fatigued and powerless to provide more support to others), depersonalization (showing a disengaged, cynical, cold, and unsympathetic attitude toward persons at work, especially those who seek help or ask for services), and feelings of low professional achievement (feeling personal and professional inadequacy and having a higher likelihood of committing errors during job tasks) (6). Many occupational groups utilize the burnout syndrome to articulate anxieties, depression, and difficulties in functioning within their professions and complex organizations. This phenomenon correlates with increased rates of job turnover, absenteeism, alcoholism, suicide, marital dysfunction, and depression across various occupations (3, 7, 8).

The relationship between these three dimensions is controversial in academic circles. Several researchers have interpreted the associations among these dimensions as resulting from an underlying causal process, but others considered the three dimensions as more or less co-occurring phenomena. Leiter and Maslach (9) argued that high levels of emotional exhaustion would lead to high levels of depersonalization, in turn, leading to low levels of personal accomplishment. An alternative view by Golembiewski (10) argued that depersonalization occurs first; emotional exhaustion would then occur in response to increasing depersonalization and decreasing personal accomplishment. Lee and Ashforth (11) proposed that elevated levels of emotional exhaustion directly evoked decreases in personal accomplishment rather than indirectly through depersonalization. Taris et al. (6) reviewed seven longitudinal studies with evidence for various causal orderings of the three burnout components, but none of the previous studies on this issue provided any convincing support for any particular causal order proposed so far. It is not clear how these three dimensions of job burnout interact with each other and how the configurations of the three burnout dimensions are organized (12).

Previous studies have not considered the heterogeneity among samples, making it difficult to draw unified conclusions. For example, Leiter investigated 122 mental health workers and reported that low levels of personal accomplishment were longitudinally associated with high levels of emotional exhaustion (13). Bakker et al. (14) found that general practitioners who reported high levels of depersonalization in the first wave of their study experienced higher job demands in the second wave. In turn, high demands were cross-sectionally associated with elevated levels of emotional exhaustion. Indeed, there are obvious individual differences in the manifestation of job burnout, as the job demands and resources perceived by different individuals vary significantly (15). However, the relationship between the three dimensions of job burnout and the distinct patterns of job burnout in Chinese police remains underexplored. Therefore, we propose hypothesis H1: There are differences in individual performance across the three dimensions of job burnout, and there are different latent subtypes of job burnout.

### 2.2 The factors associated with job burnout

Goodman was the first to study the relationships between certain biographical (16), demographic, and situational variables as they relate to burnout among police officers and to develop a model of burnout, the finding that trait anxiety is significant in relation to burnout. Over the past decade, extensive literature has emerged focusing on risk factors associated with the onset and progression of burnout syndrome. Job stress is a predictor of job burnout (8, 17, 18). Additionally, research has examined bureaucratic characteristics influencing officers’ burnout experiences (19, 20). Younger officers were found to be more susceptible to depersonalization compared to their older counterparts (2). Some studies have also explored differences between police agencies and categories (3, 21).

There is evidence indicating that burnout significantly impacts various aspects, including mental health, job performance, turnover intention (2, 22, 23), attitudes toward violence (24), and behavior in conflict situations (25). Burnout also leads to work–family conflict (26–28), emotional labor (29), and including suicide (30–33). The World Health Organization (WHO), the European Foundation for the Improvement of Living and Working Conditions, and the European Agency for Safety and Health at Work emphasize the negative consequences of job burnout (34–37).

As a special occupational group, police play a crucial role in upholding public order, combating crime, and enforcing the law (38), consequently, police performance is a critical indicator, taking the importance of the job into account. Additionally, mental health problems become rampant due to the high stress, high confrontation, and high danger of the occupation, including burnout, depression, and anxiety among police officers (8). The mental health of the police officers is also a major concern. Based on hypothesis 1, this study examines the associations among different job burnout subtypes, job performance, and mental health. Hence, we propose hypothesis H2: Different job burnout subtypes have differences in job performance and mental health.

### 2.3 The double-track human resource management model of Chinese police

In the context of increasing crime and disorder, Chinese police departments have adopted the double-track human resource management model to address the problem of manpower shortage in the police. In this model, the police forces are divided into the civilian police and the auxiliary police, with auxiliary police recruited as contract workers, distinguishing them from regular civilian police. As an auxiliary force of the civilian police, the auxiliary police face the same substantial work pressure as civilian police when dealing with complex, massive, and dangerous tasks (39). However, the auxiliary police are treated as second-class personnel within public security organizations. Compared to civilian police, the auxiliary police receive a lower salary and have lower job qualifications, yet their rank structures and training are not standardized (40). High degrees of workload and stress, lack of advancement opportunities, and low levels of compensation have led to severe job burnout and mental health problems (2, 3). Although the civilian police and the auxiliary police belong to the same police system, they differ in professional status, salary and benefits, career development, and public respect. Despite numerous studies exploring burnout among police officers, there remains a gap in the literature regarding the contribution of the double-track human resource management model, distinguishing between civilian police and auxiliary police. The study examines civilian police and auxiliary police separately, exploring their differences in job burnout, job performance, and mental health. Hence, we propose hypothesis H3: There are differences between civilian police and auxiliary police in job burnout, job performance, and mental health.

As a prevalent occupational health issue, job burnout has received attention worldwide. However, the manifestations, causes, and effects of job burnout on individuals and organizations may vary significantly across different cultural and social structural backgrounds. In China, the unique political system and sociocultural context provide a distinctive perspective for studying job burnout. Additionally, China’s social structure, values, work ethics, and organizational management methods may have a unique impact on the burnout of civilian and auxiliary police officers. Therefore, building upon the aforementioned research hypotheses, this study aims to investigate the contribution of the double-track human resource management model to burnout and mental health within frontline police departments. An in-depth study of job burnout among Chinese civilian police and auxiliary police can not only reveal the characteristics of burnout in specific cultural contexts but also provide new theoretical perspectives and empirical data for global burnout research. Additionally, it seeks to provide recommendations to police departments regarding factors that may either alleviate or exacerbate police officer burnout.

## 3 Materials and methods

### 3.1 Participants and procedures

The cross-sectional study used cluster sampling to survey all police of all 118 frontline police stations of City H, an economically underdeveloped area of China. In this survey, there are four questions designed as lie detection consistency checks. Consequently, to ensure the reliability and validity of the results, some participants who did not meet the criteria were excluded: (1) the difference between age and years of police service was greater than 18; (2) the difference between Question 14 and Question 40 (I am confident in my ability to effectively complete various tasks) was less than 2; (3) identical responses to Question 25 (I can accurately discern the other person’s emotions from their tone or facial expressions) and Question 41 (I struggle to accurately discern the other person’s emotions from their tone or facial expressions). The assessment was conducted in a quiet environment with the organization of the police officers, and participants were asked to fill in the questionnaire. Finally, a total of 889 police officers were recruited, yielding 839 valid responses (94.37% response rate), including 461 civilian police and 378 auxiliary police.

### 3.2 Measures

#### 3.2.1 Job Performance Questionnaire

The questionnaire designed by the research team comprised three dimensions: law enforcement ability, organizational ability, and communication ability, totaling 18 questions. Law enforcement ability examines the police’s proficiency in handling cases, including criminal and public security incidents, based on their mastery of relevant legal knowledge. Organizational ability examines the police’s effectiveness in achieving goals, utilizing various methods, and coordinating resources, including relationship management and manpower supervision. Communication ability examines the police’s capacity to establish effective communication with stakeholders, facilitating the exchange of ideas, emotions, and information to accomplish policing objectives. The questionnaire uses Likert-type 5-point scoring, ranging from 1 (very inconsistent) to 5 (very consistent), with higher scores indicating greater professional competence. The reliability and validity of the self-developed questionnaire were examined using partial least squares structural equation modeling (PLS-SEM), and the results indicated that the questionnaire possessed good reliability and validity (see Section 4.2).

#### 3.2.2 Job burnout questionnaire

The Maslach Burnout Inventory-General Survey (MBI-GS) by Maslach and Jackson (5) was used to measure job burnout which comprised 15 questions across three dimensions: emotional exhaustion, depersonalization, and personal accomplishment. The questionnaire uses Likert 5-point scoring, ranging from 1 (very inconsistent) to 5 (very satisfied), with higher scores indicating greater levels of job burnout. The reliability of the questionnaire was good (Cronbach α = 0.91).

#### 3.2.3 Symptom checklist questionnaire (SCL-90)

The two subscales of depression and anxiety in the symptom checklist (SCL)-90 (41) were used to examine the mental health of police officers. There are 10 questions on the anxiety scale. A higher score indicates higher levels of anxiety. The depression scale consists of 13 questions, with higher scores representing higher levels of depression. Both scales use Likert 5-point scoring to evaluate symptoms of police officers, ranging from 1 (not at all) to 5 (extremely), with higher scores indicating greater levels of depression or anxiety. In this study, Cronbach’s α test suggests that the reliability of these two scales is good: Cronbach’s α = 0.94 (anxiety) and Cronbach’s α = 0.95 (depression).

### 3.3 Ethical consideration

We ensured participants’ confidentiality and voluntary participation. The research project was examined and approved by the local Ethics Committee of the School of Psychology, South China Normal University (SCNU-PSY-2021-395), indicating that approval from a local university Institutional Review Board (IRB) was obtained. The purpose of the study was highlighted before the distribution of the questionnaire, and informed consent forms were provided to all participants. Rigorous processing was conducted on the collected and analyzed data to ensure anonymity.

### 3.4 Data analysis

Data analysis in this study utilized R 4.4 and Statistical Package for the Social Sciences (SPSS) version 21.0 software. The analysis proceeded in three steps.

First, with R 4.4, latent profile analysis (LPA) was used to explore the potential profiles of job burnout of civilian police and auxiliary police, determine the optimal fitting model, establish the number of profiles, and compare various models. A series of nested LPA models were fitted and compared to determine the best number of clusters. The process began with a one-class model and progressed by adding one additional class at each step until further improvement in model fit ceased. Model fit was evaluated using established criteria outlined by Berlin, Williams, and Parra (42), including the Akaike Information Criterion (AIC), sample size-adjusted Bayesian Information Criterion (aBIC), the Lo–Mendell–Rubin Test (LMRT), and the Bootstrapped Lo–Mendell–Rubin Test (BLMRT). Lower AIC and aBIC values indicated a better fit, while LMRT and BLMRT assessed model improvement relative to a model with *k*-1 profiles; if the *p*-value of this test was below 0.05, then the *k*-1 model should be rejected in favor of the model with more profiles. We also assessed entropy, which refers to the average accuracy in assigning people to profiles; entropy values ranged from 0 to 1, with higher values indicating greater accuracy. In addition to fit indices, each model was assessed for interpretability in light of prior theory to avoid selecting too many profiles. Finally, we also rejected models that contained small profiles (e.g., less than 1% or *n* = 25), as these profiles are typically spurious (43).

Subsequently, the Bolck, Croon, and Hagenaars (BCH) method of LPA follow-up analysis was used to ensure that the classification errors in the latent profiles were taken into account, providing more accurate and unbiased estimates.

Finally, with SPSS version 21.0 software, one-way analysis of variance (ANOVA), *t*-tests, and Chi-squared tests were applied to examine differences in job burnout profiles concerning job performance and mental health within the established model.

## 4 Results

### 4.1 Common method bias

In this study, Harman’s single-factor test was used to test the common method bias. The results showed that the variance explained by the first factor was 28.13%, which is less than the critical value of 40% (44), indicating that common method bias in this study was not significant.

### 4.2 PLS-SEM of Job Performance Questionnaire

Our study employed the PLS-SEM modeling (45, 46) to conduct dimensional validation and reliability and validity assessment of the “Job Performance Questionnaire.” As shown in Table 1, the factor loadings of the items in the Job Performance Questionnaire ranged from 0.912 to 0.678, all of which reached significance levels (*p* < 0.001). As a reliability assessment metric in PLS-SEM, the composite reliability (CR) for the three dimensions of the Job Performance Questionnaire was 0.806, 0.881, and 0.845, respectively, all exceeding the threshold of 0.7, indicating strong CR for all three dimensions. The validity of the questionnaire was examined using the “Average Variance Extracted” (AVE) and the Fornell–Larcker criterion. The AVE values for each dimension were 0.666, 0.543, and 0.684, all exceeding the threshold of 0.5 (47). The results of the Fornell–Larcker criterion are presented in Table 2. These findings demonstrate that the Job Performance Questionnaire possesses good reliability and validity.

**Table 1:** The factor loadings of items in the “Job Performance Questionnaire”.

	Law enforcement ability	Organizational ability	Communication ability
Enforcement 1	0.847		
Enforcement 2	0.912		
Enforcement 3	0.701		
Enforcement 4	0.712		
Enforcement 5	0.689		
Enforcement 6	0.820		
Enforcement 7	0.799		
Enforcement 8	0.726		
Enforcement 9	0.728		
Organizational 1		0.759	
Organizational 2		0.711	
Organizational 3		0.901	
Organizational 4		0.697	
Organizational 5		0.724	
Communication 1			0.806
Communication 2			0.892
Communication 3			0.797
Communication 4			0.785

**Table 2:** The results of the Fornell–Larcker criterion.

	Law enforcement ability	Organizational ability	Communication ability
Law enforcement ability	0.816		
Organizational ability	0.578	0.737	
Communication ability	0.298	0.580	0.827

### 4.3 Participant characteristics

Table 3 displays descriptive statistics of the participants. Among civilian police, 92% were male, with ages ranging from 26 to 30 years (22%) and over 45 (23%) years. The majority were married (80%) with a monthly income of 4,000–8,000 yuan (77%), most were socially endorsed (61%), and the distribution of their ages was relatively even. Among the auxiliary police, the majority were male (95%), with an age distribution of less than 35 years (76%), most were married (63%), earned less than 4,000 yuan per month (19%), had served for less than 12 years (96%), and were largely recruited from the community (78%).

**Table 3:** Demographics variables.

Demographics variables		Police category			
		Civilian police (*N* = 461)		Auxiliary police (*N* = 378)	
		*n*	Percentage	*n*	Percentage
Sex	Male	425	92	359	95
	Female	36	8	19	5
Age	<26 years	43	9	102	27
	26–30 years	102	22	111	29
	31–35 years	73	16	77	20
	36–40 years	70	15	64	17
	41–45 years	65	14	16	4
	>45 years	108	23	8	2
Marriage status	Spinsterhood	78	17	136	36
	Married	369	80	238	63
	Others	14	3	4	1
Monthly income	<2000 RMB	44	10	279	74
	2000–4,000 RMB	41	9	99	26
	4,000–6,000 RMB	178	39	0	–
	6,000–8,000 RMB	175	38	0	–
	>8,000 RMB	23	5	0	–
Police service years	0–1 year	78	17	182	48
	2–5 years	97	21	95	25
	6–12 years	68	15	85	22
	13–22 years	106	23	16	4
	>23 years	112	24	0	–
Recruitment mode	Police academy recruitment	76	16	2	1
	Military recruitment	23	5	16	4
	Social recruitment	283	61	293	78
	Other recruitment	79	17	67	18

RMB, renminbi (currency of China).

### 4.4 The difference test between civilian police and auxiliary police

From the results of the difference test in Table 4, there are significant differences between the civilian police and the auxiliary police. The job burnout of civilian police is higher than that of auxiliary police. The job performance of civilian police is higher than that of auxiliary police. The mental health issues among auxiliary police are more severe. Furthermore, the result of the Chi-squared test indicates that civilian police and auxiliary police exhibit significantly different levels of monthly incomes (*χ^2^* = 679.79, *p* < 0.001), with civilian police demonstrating a higher monthly outcome than auxiliary police.

**Table 4:** The result of the difference test.

Variables	Civilian police (*N* = 461)		Auxiliary police (*N* = 378)		*t*
	*M*	SD	*M*	SD	
*Job burnout*					
Emotional exhaustion	3.07	1.02	2.28	0.98	11.353***
Depersonalization	2.48	0.96	1.91	0.89	8.861***
Personal accomplishment	1.79	0.64	1.61	0.60	4.111***
*Job performance*					
Law enforcement ability	4.18	0.58	4.00	0.58	4.021***
Organizational ability	4.30	0.66	4.04	0.69	9.251***
Communication ability	4.30	0.58	4.10	0.60	7.688***
*Mental healthy*					
Anxiety	1.39	0.61	1.93	0.83	3.011***
Depression	1.40	0.66	1.93	0.83	2.969***

*p < 0.05, **p < 0.01, ***p < 0.001; SD, standard deviation.

### 4.5 The results of latent profile analysis of civilian police

The study used a 1-category model as the baseline model, and a total of 6 models were fitted. The fitting indexes are presented as shown in Table 5. Although results indicated that Model 4 possessed a better performance on fitting indexes, it did not pass the LMRT, thus Model 3, which also had a good performance, was chosen. Consequently, combined with the fitting indexes and the actual situation, the job burnout of civilian police can be divided into three subtypes.

**Table 5:** Civilian police potential profile model fitting index table.

Model	AIC	BIC	aBIC	Entropy	LMRT	BLMRT	Class probability
Model 1	20117.34	20241.34	20146.13				1.00
Model 2	18124.27	18314.41	18168.42	0.92	<0.0001	<0.0001	0.52/0.48
Model 3	**17570.79**	**17827.06**	**17630.29**	**0.89**	**0.09**	**<0.0001**	0.21/0.36/0.43
Model 4	17131.75	17454.16	17206.61	0.90	0.26	<0.0001	0.25/0.23/0.36/0.17
Model 5	16824.22	17212.76	16914.43	0.91	0.47	<0.0001	0.08/0.19/0.24/0.33/0.17
Model 6	16675.31	17129.98	16780.87	0.90	0.28	<0.0001	0.08/0.15/0.09/0.29/0.17/0.23

AIC, Akaike Information Criterion; aBIC, adjusted Bayesian Information Criterion; BIC, Bayesian Information Criterion; BLMRT, Bootstrapped Lo–Mendell–Rubin Test; LMRT, Lo–Mendell–Rubin Test. Bold indicates that the model is the best performer.

**Figure 1:**
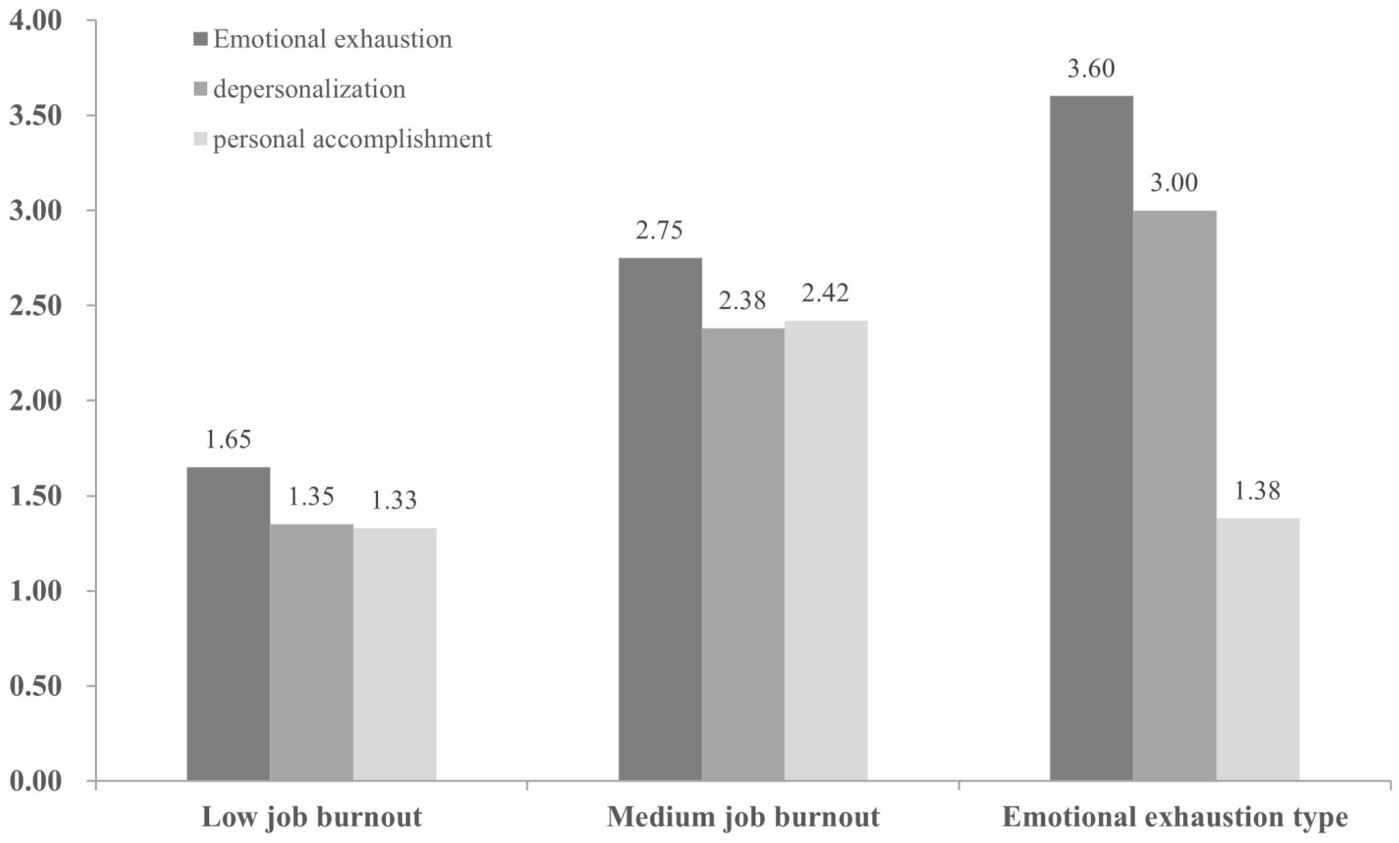
Performance of civilian police on three job burnout’s categories.

Figure 1 and Table 6 show the trend of job burnout subtypes among civilian police. The first profile, consisting of 43% of civilian police (*n* = 198), was labeled low job burnout due to relatively low levels of emotional exhaustion (*M* = 2.21, standard deviation [SD] = 0.73), depersonalization (*M* = 1.59, SD = 0.48), and personal accomplishment (*M* = 1.40, SD = 0.40). The second profile included 21% of the civilian police (*n* = 97). The results of job burnout are as follows: emotional exhaustion (*M* = 3.55, SD = 0.71), depersonalization (*M* = 3.24, SD = 0.61), and personal accomplishment (*M* = 2.75, SD = 0.39), which is called medium job burnout. Finally, the third profile was labeled emotional exhaustion type (36%, *n* = 166) due to having the highest levels of emotional exhaustion and low levels of personal accomplishment.

**Table 6:** Analysis of the differences in various dimensions of civilian police job burnout types.

	Emotional exhaustion		Depersonalization		Personal accomplishment	
	*M*	SD	*M*	SD	*M*	SD
Low job burnout	2.21	0.73	1.59	0.48	1.40	0.40
Medium job burnout	3.55	0.71	3.24	0.61	2.75	0.39
Emotional exhaustion type	3.81	0.64	3.08	0.64	1.70	0.38
*F*	267.54***		413.60***		391.76***	
Backtesting	Low < Medium < Emotional		Low < Medium = Emotional		Low < Emotional < Medium	

*p < 0.05, **p < 0.01, ***p < 0.001; SD, standard deviation.

Table 6 shows significant differences in emotional exhaustion (*F* = 267.54, *p* < 0.001) and personal accomplishment (*F* = 391.76, *p* < 0.001) among the three profiles. For depersonalization, low job burnout is significantly lower than medium job burnout and emotional exhaustion type, with no significant difference between medium job burnout and emotional exhaustion type.

### 4.6 The results of latent profile analysis of auxiliary police

The results for auxiliary police are presented in Table 7. The results indicated that Model 3, with a high entropy value of 0.94, provided the best fit, given its superior performance across various metrics. Consequently, combined with the fitting index and the actual situation, the job burnout of auxiliary police can also be divided into three subtypes.

**Table 7:** Auxiliary police potential profile model fitting index table.

Model	AIC	BIC	aBIC	Entropy	LMRT	BLMRT	Class probability
Model 1	16049.38	16167.43	16072.25	–	–	–	1.00
Model 2	14518.87	14699.88	14553.93	0.93	<0.0001	<0.0001	0.39/0.61
Model 3	**13977.14**	**14221.10**	**14024.39**	**0.94**	**0.12**	**<0.0001**	0.25/0.57/0.18
Model 4	13668.09	13975.02	13727.54	0.92	0.09	<0.0001	0.20/0.39/0.11/0.30
Model 5	13395.89	13765.77	13467.53	0.93	0.39	<0.0001	00.14/0.05/0.21/0.20/0.40
Model 6	13241.24	13674.08	13325.07	0.92	0.44	<0.0001	0.04/0.12/0.15/0.21/0.32/0.16

BLMRT, Bootstrapped Lo–Mendell–Rubin Test; LMRT, Lo–Mendell–Rubin Test. Bold indicates that the model is the best performer.

**Figure 2:**
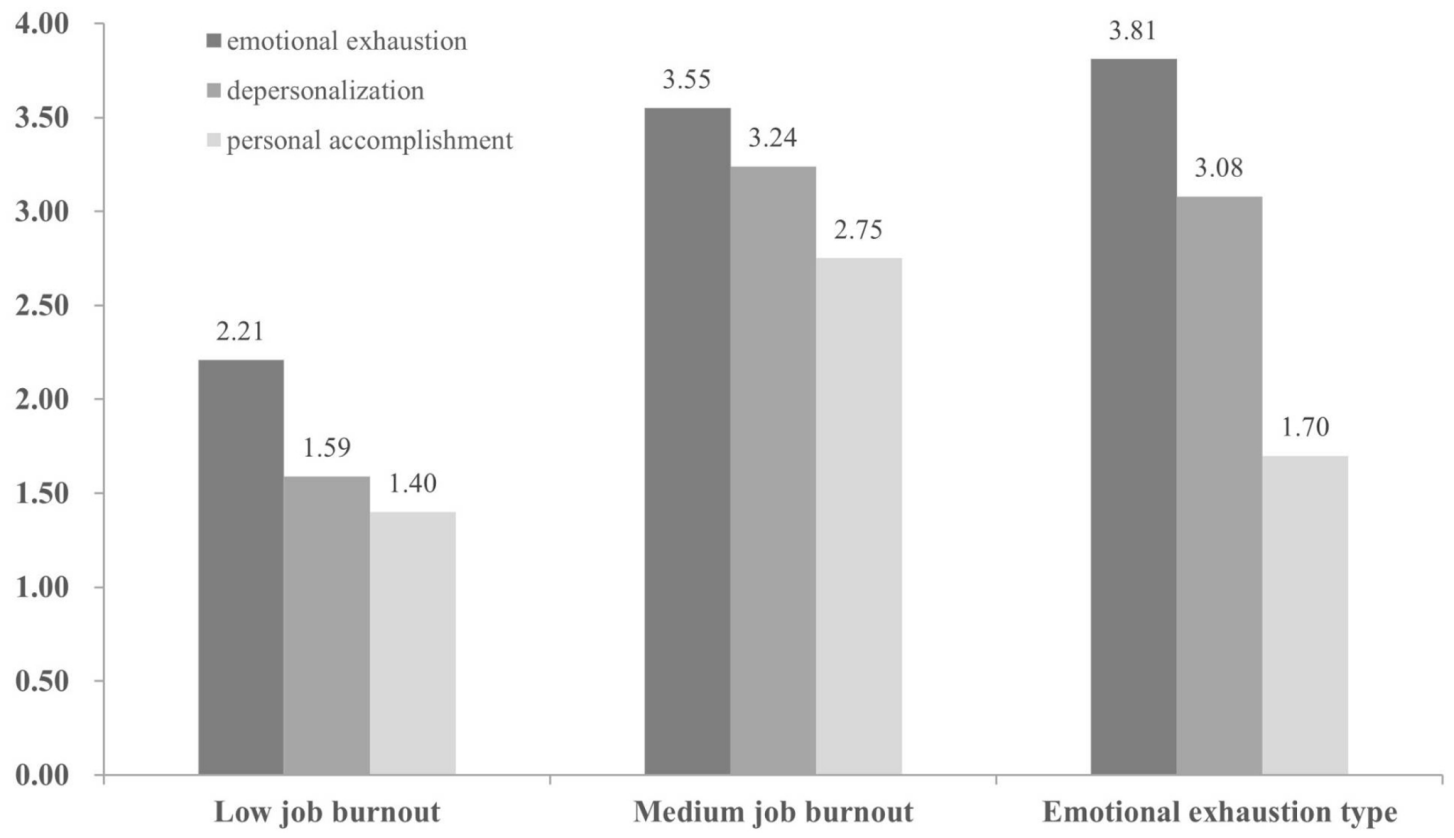
Performance of auxiliary police on three job burnout’s categories.

Figure 2 and Table 8 show the job burnout subtypes for auxiliary police. The first profile, consisting of 57% of auxiliary police, was labeled low job burnout due to relatively low levels of emotional exhaustion (*M* = 1.65, SD = 0.58), depersonalization (*M* = 1.35, SD = 0.48), and personal accomplishment (*M* = 1.33, SD = 0.37). The second profile included 25% of the auxiliary police; the results of job burnout are as follows: emotional exhaustion (*M* = 2.75, SD = 0.60), depersonalization (*M* = 2.38, SD = 0.62), and personal accomplishment (*M* = 2.42, SD = 0.42). This profile was labeled medium job burnout. Finally, the third profile was labeled the emotional exhaustion type (18%) due to having the highest levels of emotional exhaustion and a low level of personal accomplishment.

**Table 8:** Analysis of the differences in various dimensions of auxiliary police job burnout types.

Dimensions	Emotional exhaustion		depersonalization		Personal accomplishment	
	*M*	SD	*M*	SD	M	SD
Low job burnout	1.65	0.58	1.35	0.48	1.33	0.37
Medium job burnout	2.75	0.60	2.38	0.62	2.42	0.42
Emotional exhaustion type	3.60	0.66	3.00	0.82	1.38	0.33
*F*	313.12***		242.13***		292.74***	
Backtesting	Low < Medium < Emotional		Low < Medium < Emotional		Low = Emotional < Medium	

*p < 0.05, **p < 0.01, ***p < 0.001; SD, standard deviation.

In Table 8, significant differences exist between emotional exhaustion (*F* = 313.12, *p* < 0.001) and depersonalization (*F* = 242.13, *p* < 0.001) among the three profiles. In terms of personal accomplishment, low job burnout and emotional exhaustion type are significantly lower than medium job burnout, with no significant differences between low job burnout and emotional exhaustion type.

### 4.7 The relationship between police job burnout with job performance and mental health

The study investigated the association between job burnout types and job performance, as well as mental health. Job burnout type was treated as the independent variable, while job performance (comprising law enforcement ability, communication ability, and organizational ability) and mental health (anxiety and depression) were treated as dependent variables. Wald tests were conducted, correcting for classification errors. The results are presented below.

From the results in Tables 9, 10, compared to communication ability and organizational ability, civilian police perform the weakest on law enforcement ability. Regarding job performance, there is no significant difference between low job burnout and the emotional exhaustion type, both are significantly better than medium job burnout. In terms of mental health, there are significant differences among the three profiles (*F*_anxiety_ = 81.30, *F*_depression_ = 62.35), with medium job burnout being more severe.

**Table 9:** The descriptive statistics of civilian police.

Variables	Low job burnout		Medium job burnout		Emotional exhaustion type	
	*M*	SD	*M*	SD	*M*	SD
Law enforcement ability	4.44	0.31	3.48	0.39	4.35	0.34
Communication ability	4.53	0.35	3.64	0.30	4.52	0.37
Organizational ability	4.61	0.33	3.54	0.34	4.40	0.31
Anxiety	1.14	0.35	1.93	0.39	1.62	0.33
Depression	1.17	0.30	1.82	0.39	1.58	0.40

SD, standard deviation.

**Table 10:** The difference test of civilian police.

Variables	Global	Low job burnout vs. Medium job burnout	Medium job burnout vs. Emotional exhaustion type	Low job burnout vs. Emotional exhaustion type	Multiple comparison
Law enforcement ability	288.03***	281.16***	130.88***	1.78	Low = Emotional > Medium
Communication ability	215.11***	200.61***	141.53***	0.01	Low = Emotional > Medium
Organizational ability	219.61***	219.61***	84.36***	2.15	Low = Emotional > Medium
Anxiety	81.30***	37.35***	5.03*	47.93***	Low < Emotional < Medium
Depression	62.35***	29.35***	3.51*	36.64***	Low < Emotional < Medium

*p < 0.05, **p < 0.01, ***p < 0.001.

From the results of Tables 11, 12, there are significant differences in job performance among the three profiles (*F*_law_ = 347.95, *F*_communication_ = 277.42, *F*_organization_ = 316.21), indicating that low job burnout is better than the emotional exhaustion type, and both are better than medium job burnout. In terms of mental health, there are significant differences among the three profiles (*F*_anxiety_ = 225.45, *F*_depression_ = 184.48), and the emotional exhaustion type is more severe.

**Table 11:** The descriptive statistics of auxiliary police.

Variables	Low job burnout		Medium job burnout		Emotional exhaustion type	
	*M*	SD	*M*	SD	*M*	SD
Law enforcement ability	4.34	0.41	3.33	0.45	4.02	0.38
Communication ability	4.38	0.39	3.41	0.43	4.20	0.42
Organizational ability	4.39	0.44	3.25	0.40	4.11	0.41
Anxiety	1.39	0.23	2.21	0.20	2.54	0.22
Depression	1.43	0.27	2.18	0.22	2.50	0.25

SD, standard deviation.

**Table 12:** The difference test of auxiliary police.

Variable	Global	Low job burnout vs. Medium job burnout	Medium job burnout vs. Emotional exhaustion type	Low job burnout vs. Emotional exhaustion type	Multiple comparison
Law enforcement ability	347.95***	137.86***	345.50***	35.65***	Low > Emotional > Medium
Communication ability	277.42***	146.76***	267.12***	10.93***	Low > Emotional > Medium
Organizational ability	316.21***	152.00***	303.87***	17.57***	Low > Emotional > Medium
Anxiety	225.45***	7.68**	132.89***	118.60***	Low < Medium < Emotional
Depression	184.48***	7.00**	113.87***	89.74***	Low < Medium < Emotional

*p < 0.05, **p < 0.01, ***p < 0.001.

## 5 Discussion

The research results show that the double-track human resource management model contributes to job burnout, job performance, and the mental health of police officers. There are three subtypes of job burnout among Chinese police, and between civilian police officers and auxiliary police officers, the main types of job burnout differ significantly, along with differences in mental health and job performance. Civilian police exhibit more severe job burnout, with emotional exhaustion being predominant, which subsequently contributes to their mental health. Moreover, due to the greater resources available to civilian police, their job performance tends to be higher.

### 5.1 The contribution of double-track human resource management to job burnout and mental health among Chinese police officers

In China, the biggest problem with the double-track human resource management model is that it weakens the sense of competition. Civilian police, as permanent workers, have an ‘iron rice bowl’; they enjoy higher pay even if they do not work hard and do not worry about getting fired (48, 49), leading to poor work enthusiasm and job burnout among civilian police in the long run. Engel et al. (50) demonstrated that emotional exhaustion was reported to be higher in officers with a longer length of service and at a middle career stage (compared with an upper-middle career stage). Our research reveals that civilian police, with the characteristic of predominantly over 30 years old and stable incomes, experience heightened job burnout due to years of repetitive tasks, while auxiliary police, with the characteristic of mostly under 35 and less than 5 years of service, exhibit lower job burnout, which likely results from auxiliary police’s younger age and shorter tenure.

LPA generated three burnout profiles among police in China: low job burnout, medium job burnout, and emotional exhaustion type. These results support previous research studies conceptualizing burnout as a complex, multidimensional phenomenon in which the three burnout dimensions manifest differently in individuals (7, 51–58). Leiter and Maslach (51) argued that high levels of emotional exhaustion would lead to high levels of depersonalization in turn leading to low levels of personal accomplishment. However, our research reveals that police experiencing emotional exhaustion show severe depersonalization, as well as higher levels of achievement. This indicates that achievement is a relatively independent dimension and a key factor affecting police job performance. Prior meta-analytic studies have supported that each of these three components shares unique associations with various determinants and outcomes (11). Emotional exhaustion is typically considered the central aspect of burnout and its most overt manifestation (6). Leiter and Maslach (9) argued that high levels of emotional exhaustion predict high levels of depersonalization, and emotional exhaustion was positively related to depersonalization [as shown in the studies by Leiter and Maslach (9) and Golembiewski et al. (59)], but where personal accomplishment developed independently from depersonalization. Cordes and Dougherty (60) argued that feelings of personal accomplishment are better conceptualized as a personality factor rather than as a burnout component. Our research has come to the same result and also found that achievement is the main factor contributing to job performance.

Compared to the general working population, police personnel are twice as likely to attribute their mental health issues to work-related problems, and some studies suggested that burnout and depressive symptoms seem to cluster together and develop in parallel (61, 62). Under the double-track human resource management model, we found that the factors influencing the mental health of civilian police and auxiliary police differ. Achievement affects the mental health of civilian police, while emotional exhaustion affects that of auxiliary police. This is directly related to the imperfect management of auxiliary police. In China, this group has become a scapegoat for violent law enforcement, forced demolition, corruption, and other incidents used to evade laws and social responsibilities. This has led to negative public stereotypes, such as “abusers,” “low quality,” and “backstabbers.” These psychosocial risk factors uniquely impact police officers’ burnout (63). In fact, due to the strong correlation between the public establishment of auxiliary police and “low quality,” many auxiliary police have a negative self-perception of their occupation and lack a sense of belonging. Many auxiliary police internalize this negative image, experiencing a sense of inadequacy and a lack of recognition from their organization (20), ultimately leading to significant mental health challenges.

This study not only supports previous findings on emotional exhaustion, depersonalization, and personal accomplishment but also deepens the understanding of the multidimensional nature of job burnout.

### 5.2 Recommendations of the research for the double-track human resource management

Due to the particularity of China’s political system, significant differences exist between civilian police and auxiliary police, primarily due to their respective job roles and management structures (64, 65). Some studies suggested that job stress and coping are significantly correlated with subjective wellbeing (66, 67), which is also predicted by perceived social support, perceived organizational support, and perceived control of internal states (68, 69). Therefore, the double-track human resource management model is disadvantageous to auxiliary police in these aspects. This study reveals heightened job burnout among civilian police, juxtaposed with lower professional proficiency and mental wellbeing among auxiliary police. According to this study, achievement is a relatively independent dimension and a key factor affecting the job performance and mental health of civilian police, while emotional exhaustion primarily affects the mental health of auxiliary police. Therefore, the double-track human resource management model is disadvantageous to auxiliary police in these aspects and needs adjustments to reduce job burnout among civilian police, improve the mental health of auxiliary police, and enhance the overall quality and performance of the police workforce.

To resolve the potential risks associated with the double-track human resource management model in public security organs, adjustments are needed in both management and treatment distribution systems. First, personnel management within public security organs should transition from identity-based management to contract and position-based management. The disparity in treatment between on-duty and off-duty personnel stems from traditional identity management. Thus, it is imperative to transcend these constraints, unify employment and contract systems, and integrate off-duty personnel. This shift eliminates establishment status constraints, aligning human resource management with market economy principles. Second, adhering to the principle of position-based compensation and merit-based distribution, each position should have a salary commensurate with its responsibilities and functions. Implementing competitive employment principles ensures dynamic adjustments and opportunities for advancement or demotion based on job performance. Competitors vie for positions based on job criteria, independent of personal identity or qualifications, promoting fairness and just compensation practices and rectifying disparities in pay and distribution. Additionally, adequate mental health support is necessary. These measures are crucial for improving the overall wellbeing and job performance of all police officers and fostering a more motivated, healthy, and effective police force capable of better serving the public and maintaining law and order.

### 5.3 Limitations

First, the study sample is influenced by the characteristics of the personnel and the region. The sample for this research was drawn from an economically underdeveloped area in China, and differences in economic development across regions may impact police welfare and salaries. Therefore, caution should be exercised when generalizing the results. Second, this study is essentially a cross-sectional correlational study. The conclusions regarding the differential contribution of the double-track human resource management model to the job burnout and mental health of auxiliary and civilian police should be interpreted with caution. Future research could consider incorporating police officers from a broader range of regions to enhance the generalizability and representativeness of the findings. Additionally, longitudinal studies should be conducted to track changes in job burnout and mental health throughout police careers, offering a more comprehensive understanding of the long-term contributions of these issues.

## 6 Conclusion

Our study reveals significant disparities between Chinese civilian police and auxiliary police. However, job burnout among both civilian police and auxiliary police can be divided into three subgroups: the “low job burnout” group, the ‘medium job burnout’ group, and the “emotional exhaustion type” group. Police officers with different types of job burnout differ in their levels of mental health and job performance. Among the civilian police, the “medium job burnout” group exhibits the poorest job performance and the most severe mental health problems. Among the auxiliary police, the “medium job burnout” group exhibits the poorest job performance, and the “emotional exhaustion type” group exhibits the most severe mental health problems. This study reveals the characteristics of job burnout among Chinese police officers, suggesting that we should promptly identify individuals with high levels of job burnout and implement targeted intervention measures to enhance mental health and job performance.

## Data availability statement

The datasets presented in this article are not readily available because the data involves government departments concerns and the permissions do not allow the authors to disclose the original data. The data cannot be disclosed without the updated permission of the government departments. Requests to access the datasets should be directed to Minqiang Zhang, 2640726401@qq.com.

## Ethics statement

The studies involving humans were approved by the local Ethics Committee of the School of Psychology, South China Normal University (SCNU-PSY-2021-395). The studies were conducted in accordance with the local legislation and institutional requirements. The participants provided their written informed consent to participate in this study.

## Author contributions

ZL: Conceptualization, Data curation, Formal analysis, Investigation, Methodology, Project administration, Software, Visualization, Writing – original draft, Writing – review & editing. MiY: Formal analysis, Validation, Writing – review & editing. HL: Formal analysis, Validation, Writing – review & editing. JC: Formal analysis, Validation, Writing – review & editing. MeY: Validation, Writing – review & editing. TT: Validation, Writing – review & editing. HY: Validation, Writing – review & editing. YZ: Validation, Writing – review & editing. MZ: Conceptualization, Funding acquisition, Methodology, Project administration, Resources, Supervision, Validation, Writing – review & editing.
